# Health Equity Considerations for Children With Type 1 Diabetes Who Utilize Insulin Cost-Sharing Cap Programs

**DOI:** 10.7759/cureus.63588

**Published:** 2024-07-01

**Authors:** Katie DeWitt, Petter Bjornstad, Kelly R Bergmann

**Affiliations:** 1 Pediatric Emergency Medicine, Children's Minnesota, Minneapolis, USA; 2 Medicine, University of Washington (UW) Medicine Diabetes Institute, Seattle, USA

**Keywords:** type 1 diabetes, diabetes mellitus, healthy equity, public heath, medicaid population

## Abstract

The cost associated with type 1 diabetes care is considerable and the rising price of insulin has further amplified this financial burden. To curb insulin costs, numerous policies have been enacted in the past five years, both by pharmaceutical companies and their intermediaries, as well as federal and state legislatures. The most notable example is implementation of insulin cost-sharing cap laws, which place limits on out-of-pocket expenses for insulin, and in some cases, diabetes technology. Although insulin cost-sharing cap laws have the potential to mitigate the financial burden associated with routine diabetes care, these policies have largely benefitted adults living with type 1 diabetes, while children, especially those from racial and ethnic underrepresented groups, appear to have derived limited advantage. We describe the current state of insulin cost-sharing cap laws and utilization among children and adolescents with type 1 diabetes, with a focus on the limitations of current insulin laws, the importance of measuring health outcomes for children who utilize such programs, and the impact on health equity. We provide a call to action for policymakers and provide recommendations for future research in this area.

## Editorial

The cost associated with type 1 diabetes (T1D) care is considerable and the rising price of insulin has further amplified this financial burden [1]. To curb insulin costs, numerous policies have been enacted in the past five years, both by pharmaceutical companies and pharmacy benefit managers (PBMs), as well as federal and state legislatures. However, these policies have largely benefitted adults living with T1D, while children, especially those from racial and ethnic underrepresented groups, appear to have derived limited advantage.

Amid rising drug prices, manufacturers have attempted to mitigate insulin costs. Eli Lilly, Novo Nordisk, and Sanofi, the manufacturers responsible for 90% of insulin production, have all reported price cuts for insulin, along with price caps for those with commercial insurance and rebates for insurance providers. Despite these efforts, substantial cost reductions for patients have not materialized. One explanation is that the share of expenditures retained by PBMs, which act as an intermediary between manufacturers, pharmacies, and insurance companies to negotiate pricing, has been increasing. Because PBMs receive reimbursement through manufacturer rebates, they have an incentive to advocate for more expensive drugs on formularies. This may be detrimental for patients with large copayments or insufficient insurance coverage and has led policymakers to advocate for greater transparency around the complex system of pharmaceutical spending. The American Diabetes Association has recommended four specific reforms: 1) 100% rebate pass-through for insulin, effectively bypassing PBMs, 2) limiting restrictive tiering of insulin, 3) expanding rebates to include diabetes technology, and 4) limiting step therapy, where older medications must be used first before newer drugs are covered [2].

Legislative efforts have focused on the adoption of insulin cost-sharing cap initiatives. At the federal level, the Affordable Insulin Now Act was implemented in 2023 and restricts insulin copayments to $35 per month, although this is limited to Medicare beneficiaries [3]. Similar laws have been implemented at the state level, with over 25 states and the District of Columbia enacting insulin cost-sharing caps since 2019 (Table 1) but are largely restricted to those with commercial insurance. Along with Colorado, Minnesota is the only other state that includes uninsured individuals in their insulin cost-sharing cap law. Given the nascence of insulin cost-sharing cap programs, knowledge of whether such efforts lead to cost savings or improved health outcomes is lacking.

**Table 1:** States with insulin copay cap programs and implementation dates All policies cover patients with state-regulated commercial insurance. Only MN and UT also include uninsured. a Expanded in Jan 2022 to include $35 or $50 caps for select groups. b Cap of $100 for supplies. c Legislation signed but not effective until Jan 2025.

Effective Date	State	Copay/30-d supply
Jan 2020	CO	$100 ^a^
July 2020	MN	$35
Sep 2020	NH	$30
Jan 2021	CT	$25 ^b^
Jan2021	DE	$100
Jan 2021	IL	$100
Jan 2021	ME	$35
Jan 2021	NM	$25
Jan 2021	NY	$100
Jan 2021	UT	$30
Jan 2021	VA	$50
Jan 2021	WA	$35
Jul 2021	WV	$100
Sep 2021	TX	$25
Oct 2021	AL	$100
Nov 2021	OK	$30
Jan 2022	DC	$30
Jan 2022	KY	$30
Jan 2022	OR	$75
Jan 2022	VT	$100
Jan 2023	MD	$30
Jan 2023	RI	$40
Jan 2023	ND	$25
Jan 2023	LA	$75
Jan 2024	MT	$35
Jan 2024	NE	$35
Jan 2025	NJ	$30^ c^

Implications for pediatrics

Knowledge of the direct impact of insulin cost-sharing cap programs for children and adolescents is sparse. One study estimated that a $25 insulin cost-sharing cap would reduce annual out-of-pocket spending by $480, benefitting all children with T1D and a greater proportion of children enrolled in high-deductible health plans [4]. However, this study was conducted when cost-sharing cap programs were not in existence, and actual cost savings were not reported. Furthermore, laws such as the Affordable Insulin Now Act do not include coverage for children, who are typically insured through commercial plans, Medicaid, or the Children’s Health Insurance Program (CHIP) and may be eligible to receive insulin at little to no cost through prescription drug discount and rebate programs. Although discounts offset insulin prices significantly, the offset has been disproportionally attributed to commercial discounts, which increased by 2.5% from 2012 to 2019, a difference of $13 billion, whereas Medicaid rebates decreased by 9% over the same period [5].

Current eligibility requirements for Medicaid and CHIP are restrictive, and many families are left with sub-optimal coverage. For example, using the mean Medicaid eligibility level of 190% of the 2023 federal poverty level, a family of 4 must have an annual gross income of less than $57,000 in order to qualify. One potential solution to improve insulin access is to build upon Medicaid expansion, which has been adopted in 41 states. Benefits have included provisions for young adults to stay on their parent’s coverage up to age 26 and alignment of age and eligibility limits for Medicaid and CHIP. However, results from studies that have examined diabetes-related outcomes associated with Medicaid coverage have been conflicting. Among children with established T1D, public insurance has been associated with a greater likelihood of hospitalization compared to youth with private insurance, suggesting that children with public healthcare coverage may experience worse outcomes [6]. Furthermore, for every 10% increase in census tract poverty level, the odds of hospitalization for diabetic ketoacidosis (DKA) increased by 22%, and importantly, race was not associated with hospitalization [6]. On the other hand, state-level Medicaid expansion has been associated with improved access to diabetes care and lower hemoglobin A1c levels [7]. But even if outcomes data for patients with diabetes were more robust, given the political climate, it seems unlikely that there would be bi-partisan support for Medicaid expansion. Perhaps a more appealing option may be to expand insulin cost-sharing programs, which are narrower in focus, to include coverage for patients with Medicaid or CHIP.

Understanding the relationship between insulin cost-sharing programs and pediatric outcomes is essential. Children with T1D are at risk for a number of comorbidities, including DKA, which is the most common complication in children with T1D [8]. Insulin omission has been frequently associated with DKA, underscoring the importance of maintaining a continuous supply of insulin. Children with DKA are also at risk of developing other comorbid conditions, such as acute kidney injury and cerebral edema. Recent estimates suggest that approximately 50% of children hospitalized for DKA present with acute kidney injury [9]. Mitigating such comorbidities is important not only for immediate health benefits, but also for the long-term health of children living with T1D. DKA-related acute kidney injury has been associated with persistent and moderate albuminuria [9], which places patients at risk of developing diabetic kidney disease. Both cerebral edema and acute kidney injury have been associated with reduced cognitive function [10].

Considerations for health equity

The implementation of current insulin cost-sharing cap programs has sparked concerns about the equitable allocation of healthcare resources, notably affecting racial and ethnic underrepresented groups who are disproportionately represented in Medicaid and CHIP enrollments. Additionally, uninsured children are also left without access to affordable insulin under most state cost-sharing cap programs. This is significant, given that 5% of children and adolescents in the U.S. are uninsured, which represents over 4 million people. While the estimated number of uninsured non-Hispanic White and Black children are similar at 4.4% and 4.7%, respectively, the number of uninsured children who identify as Hispanic/Latino is nearly double, at 8.6% [11]. By excluding coverage for uninsured children or those with Medicaid, it is likely that insulin cost-sharing cap programs further marginalize racial and ethnic underrepresented groups. Coupled with lower rates of commercial insurance among non-Hispanic Black patients, the pre-existing disparities in T1D care may be further exacerbated by insulin cost-sharing cap programs. Non-Hispanic Black and Hispanic children more often require hospitalization for diabetes complications, are less likely to use diabetes technology use and have worse glycemic control, compared to non-Hispanic white children [12].

Conclusions

Access to affordable insulin is critical for the health of children and adolescents living with T1D. Although recent legislation and efforts from drug manufacturers have likely led to lower out-of-pocket cost for insulin, there is more work that can be done to protect vulnerable population, including children and racial and ethnic minority populations. Coupled with modifications to current insulin cost-sharing laws, researchers should study the impact of such laws on measurable health outcomes.

Recommendations

Insulin cost-sharing cap laws need to consider inclusion of all coverage types applicable to children and adolescents, including those who are uninsured. Policy makers should work towards more transparent pricing among drug manufacturers and PBMs, and on improving access to outpatient prescription drug rebates and discounts for children enrolled in Medicaid or CHIP. Future research efforts should concentrate on identifying children and adolescents who utilize insulin cost-sharing programs, and assessing measurable outcomes, including actual costs, prescription fills, and acute care utilization (e.g. emergency department visits or hospitalizations). Researchers should also seek to address whether cost-sharing cap programs are being utilized in an equitable manner, leading to further marginalization of racial and ethnic minority children. 

## References

[1] Biniek JF, Johnson W (2022). Spending on Individuals With Type 1 Diabetes and the Role of Rapidly Increasing Insulin Prices. https://healthcostinstitute.org/images/easyblog_articles/267/HCCI-Insulin-Use-and-Spending-Trends-Brief-01.22.19.pdf.

[2] (2023). PBM Policies and Their Impact on Drug and Device Costs. https://diabetes.org/tools-resources/managing-diabetes-costs/pbm-policies-and-their-impact-drug-and-device-costs#:~:text=Many%20PBMs%20have%20the%20power%20to%20determine%20what,their%20insurance%2C%20and%20therefore%20on%20patients%E2%80%99%20out-of-pocket%20costs.

[3] (2023). Text - S.3700 - 117th Congress (2021-2022): Affordable Insulin Now Act. https://www.congress.gov/bill/117th-congress/senate-bill/3700/text.

[4] Chua KP, Lee JM, Conti RM (2021). Potential change in insulin out-of-pocket spending under cost-sharing caps among pediatric patients with type 1 diabetes. JAMA Pediatr.

[5] Dickson SR, Gabriel N, Gellad WF, Hernandez I (2023). Assessment of commercial and mandatory discounts in the gross-to-net bubble for the top insulin products from 2012 to 2019. JAMA Netw Open.

[6] Maxwell AR, Jones NY, Taylor S (2021). Socioeconomic and racial disparities in diabetic ketoacidosis admissions in youth with type 1 diabetes. J Hosp Med.

[7] Pihoker C, Braffett BH, Songer TJ (2023). Diabetes care barriers, use, and health outcomes in younger adults with type 1 and type 2 diabetes. JAMA Netw Open.

[8] Tieder JS, McLeod L, Keren R (2013). Variation in resource use and readmission for diabetic ketoacidosis in children's hospitals. Pediatrics.

[9] Huang JX, Casper TC, Pitts C (2022). Association of acute kidney injury during diabetic ketoacidosis with risk of microalbuminuria in children with type 1 diabetes. JAMA Pediatr.

[10] Ghetti S, Kuppermann N, Rewers A (2020). Cognitive function following diabetic ketoacidosis in children with new-onset or previously diagnosed type 1 diabetes. Diabetes Care.

[11] Alker J, Osario A, Park E (2023). Number of Uninsured Children Stabilized and Improved Slightly During the Pandemic. https://ccf.georgetown.edu/wp-content/uploads/2022/12/Georgetown-CCF-Kids-Coverage-Report-2022.pdf.

[12] Lipman TH, Smith JA, Patil O, Willi SM, Hawkes CP (2021). Racial disparities in treatment and outcomes of children with type 1 diabetes. Pediatr Diabetes.

